# Association between dysregulated expression of Ca^2+^ and ROS-related genes and breast cancer patient survival

**DOI:** 10.3389/fbinf.2025.1633494

**Published:** 2025-09-22

**Authors:** Sofia Ramos, João Gregório, Ana Sofia Fernandes, Nuno Saraiva

**Affiliations:** 1 CBIOS, Universidade Lusófona’s Research Center for Biosciences and Health Technologies, Lisbon, Portugal; 2 Departamento de Ciencias Biomédicas, Universidad de Alcalá de Henares, Alcalá de Henares, Madrid, Spain

**Keywords:** breast cancer, gene expression, reactive oxygen species, calcium (Ca^2+^), patient survival

## Abstract

The intricate interplay between Ca^2+^ and reactive oxygen species (ROS) signalling systems influences numerous cellular pathways. Dysregulated expression of genes associated with Ca^2+^ and ROS homeostasis can significantly impact cancer progression. Despite extensive research, various underlying mechanisms remain elusive, lacking a comprehensive unified perspective. Breast cancer (BC) remains the leading cause of cancer-related deaths among women, highlighting the pressing need to discover novel regulatory mechanisms, therapeutic targets, and potential biomarkers. In this study, we employed a bioinformatic approach based on data from The Cancer Genome Atlas to assess the association between combined dysregulation of specific pairs of genes involved in redox- or Ca^2+^-related cellular homeostases and patient outcome. These genes were selected by differences in their expression between normal and tumour tissues and in their individual association with patient survival rates. Cumulative proportion survival at the 5-year post-diagnosis was calculated for each quartile of expression within the population exhibiting either high or low expression of a second gene. Additional genes with expression positively or negatively correlated with the set of relevant gene pairs were identified, and a gene enrichment analysis was performed. Our results show that the simultaneous dysregulation of a selected number of gene pairs is substantially associated with BC patient survival. Notably, the expression dysregulation of these gene pairs is associated with altered expression of genes linked to cell cycle regulation, cell adhesion, and cell projection processes. This approach exhibits a significant potential to identify new prognostic biomarkers or drug targets for BC.

## 1 Introduction

Breast cancer (BC) is the most prevalent form of cancer in women, accounting for an estimated 2.3 million new cases each year (approximately 11.7% of total cancer cases) and causing 685.000 deaths worldwide (Bray et al., 2024). The high incidence of BC has driven the development of standardized diagnostic and therapeutic guidelines (Ren et al., 2022; Kerr et al., 2022). The precise molecular mechanisms underlying BC initiation and progression remain incompletely understood. Evidence suggests that breast tumor tissues exist in a pro-oxidative environment, influenced by oestrogen-induced reactive oxygen species (ROS) and surrounding adipose tissue, contributing to tumorigenic processes (Kalezic et al., 2021; Kim et al., 2020).

ROS are natural by-products of cellular processes, primarily generated by NADPH oxidases (NOXs) and mitochondrial oxidative phosphorylation (Forrester et al., 2018). Antioxidant enzymes across cellular organelles and the extracellular space regulate their levels (Snezhkina et al., 2019). Like in other solid tumours, ROS and oxidative stress exhibit a paradoxical role in BC, contributing to both tumor progression and suppression (Halliwell, 2007). At physiological levels, ROS regulate key cellular functions, such as proliferation, apoptosis, differentiation, migration, and angiogenesis (Halliwell, 2007; Sabharwal and Paul, 2014). Breast cancer cells elevate ROS through metabolic alterations, gene mutations, and hypoxia adaptation (Huang et al., 2021; Shi et al., 2024; Perillo et al., 2020), activating redox-sensitive pathways (Lien et al., 2016; Azzam et al., 2008) to promote survival and progression. Transcription factors like NF-κB and Nrf2 upregulate antioxidant enzymes, allowing tumor cells to maintain ROS at pro-tumorigenic levels while avoiding lethal oxidative damage (Gorrini et al., 2013).

To counteract excessive oxidative stress, BC cells upregulate antioxidant enzymes such as superoxide dismutases (SOD), glutathione peroxidases (GPX), thioredoxins (TXN), peroxiredoxins (PRDX), and catalase, maintaining ROS at levels that favor survival and proliferation (Perillo et al., 2020). However, if ROS levels exceed the adaptive threshold, oxidative stress may induce senescence or apoptosis, underscoring the paradoxical role of ROS as both tumor promoters and potential suppressors (Harris et al., 2015).

Calcium ion (Ca^2+^)-dependent signalling pathways also play an important role in BC development by regulating proliferation, apoptosis evasion, angiogenesis, hypoxia adaptation, and metastasis (Prevarskaya et al., 2014). The remodelling of intracellular Ca^2+^ homeostasis observed in cancer cells can result from the up- or downregulation of Ca^2+^ channels, exchangers, pumps, and stores, affecting tumor progression by modulating cell proliferation, migration, or angiogenesis (Marchi and Pinton, 2016; Déliot and Bruno, 2015).

Additionally, Ca^2+^ and ROS interact bidirectionally, with Ca^2+^ influencing ROS production via mitochondrial metabolism and antioxidant enzyme regulation, while ROS can modulate Ca^2+^-dependent signaling (Hempel and Mohamed, 2017). This intricate interplay provides advantages to cancer cells, inhibiting apoptosis pathways while promoting tumorigenic processes such as inflammation, cell proliferation, and invasion (Nicolo et al., 2023; Hempel and Mohamed, 2017; Delierneux et al., 2020; Pathak et al., 2025; Reczek and Chandel, 2018).

Despite extensive research in these fields in recent years, the precise mechanisms involved in specific types of cancer remain largely unknown. Furthermore, several aspects of BC biology, particularly a comprehensive understanding of redox and Ca^2+^ signalling roles and their interactions in BC progression, remain poorly elucidated. Therefore, a more profound comprehension of the contribution of the mechanisms underlying ROS and Ca^2+^ signalling pathways for BC is imperative for developing novel therapeutic targets and more effective strategies for BC management. In this study, a bioinformatic approach was employed to explore the association between the co-dysregulation of genes involved in redox and Ca^2+^ homeostasis and BC patient survival. Associations between the selected genes and tumour progression biological processes were also identified.

## 2 Methods

### 2.1 Gene selection criteria

To identify genes involved in BC progression and patient survival, a PubMed exhaustive search was conducted using the following keywords and combinations: “Breast cancer AND redox”, “Breast cancer AND calcium”, “Breast cancer AND survival AND redox genes”, “Breast cancer AND survival AND calcium genes”, “Breast cancer genes AND redox AND progression”, “Breast cancer genes AND calcium AND progression”, “Breast cancer genes AND patients survival”, “Breast cancer AND redox regulation”, “Breast cancer AND calcium regulation”, “Breast cancer AND redox AND migration”, “Breast cancer AND calcium AND migration”, “Breast cancer AND redox AND proliferation”, “Breast cancer AND calcium AND proliferation” and “Breast cancer AND redox genes AND calcium genes”. Only genes supported by evidence based on human breast cancer models, such as cell lines, xenografts, and human tissues, were considered. Two sets of 39 redox- and 47 Ca^2+^-related genes were selected from published studies available in PubMed that associate these genes with BC progression and/or patient survival (Supplementary Table S1). For the selected genes, The Cancer Genome Atlas (TCGA) Breast Invasive Carcinoma collection dataset (BRCA) (composed of 1,098 patients) was used as a data source for BC gene-expression (mRNA) (Liu et al., 2018). To analyse TCGA data, the UALCAN (http://ualcan.path.uab.edu/) and the Gene Expression Profiling Interactive Analysis 2 (GEPIA2; http://gepia2.cancer-pku.cn/) platforms were used.

The relation between gene expression level and the overall patient survival was analysed using median and quartile cut-offs in GEPIA2 (Tang et al., 2019). The Log-rank test and hazard ratio (high) values with statistical differences between curves were calculated. Genes with significant differences (log rank, p < 0.05) between curves using at least one of the cut-off groups (median or quartiles) and with HR ≤ 0.8 and ≥0.2 were selected for further analysis. UALCAN (Chandrashekar et al., 2017) was used to determine differential gene expression between normal and BC tissues. Genes with statistically significant different expression (Mann-Whitney U test, p < 0.001) were selected. Additionally, supported by extensive previously published research (Supplementary Table S2), a group of redox and Ca^2+^ signalling-related genes were also included despite not having a significant expression dysregulation at the mRNA level: *LOXL2*, *NOX4*, *SOD2*, *ORAI1*, and *STIM1* (Supplementary Table S1). Since there is a strong suggestion of the impact of the proteins encoded by these genes in BC progression, their inclusion in the combination analysis could potentially identify synergistic effects.

### 2.2 Association between expression of gene pairs and BC patient survival

The relation between the expression of combinations of each two genes (defined as “pairs” from now on) and patient survival was analysed. The number of survival days until the last follow-up, status (alive/dead), and the expression levels (RSEM RNA-SeqV2) of selected genes were collected for each patient using OncoLnc (www.oncolnc.org) (Anaya, 2016). Expression levels of selected genes were categorized into quartiles (Q1-Q4). Kaplan-Meier survival analysis and Log-rank test were performed to assess the differences between patient survival curves generated based on the expression level of each gene. Survival analysis was calculated, defining death as the event of interest, quartiles of expression of one gene (Gene 1) as a factor, and quartiles of the other (Gene 2) as strata. Cumulative survival proportion and survival time were used to perform risk analysis, defining 5 years as a prognosis time point. The same process was used in the opposite direction, i.e., Gene 2 as factor and Gene 1 as strata. For this study, the confidence level was defined at 95% (p < 0,05). Statistical Package for Social Science for Windows (SPSS; v.27) was the statistical software used. Graphics showing the cumulative survival proportion of the combination between Gene 1 and Gene 2 quartiles for 5 years were drawn. The 46 gene pairs showing the same direction of 5-year survival change in both stratifications (gene A as factor, gene B as strata and *vice versa*) and exhibiting an absolute HR difference of at least 10% between the lowest and highest expression quartiles, were selected.

### 2.3 Gene enrichment analysis

**FIGURE 1:**
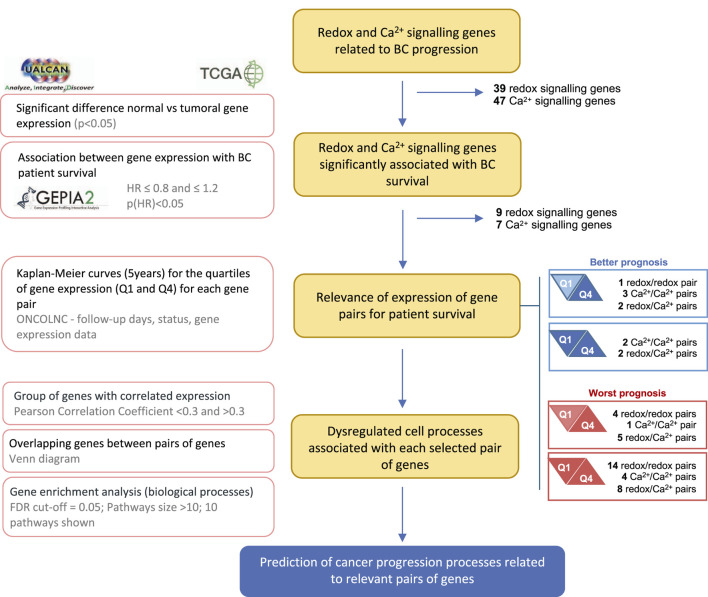
Research workflow. Flowchart showing the data sources, methodology, and criteria for gene selection. The main methodology key points are described on the left.

Using the UALCAN platform, Pearson correlations were performed to identify genes with positively or negatively correlated expression with that of selected gene pairs. Only genes with Pearson correlation coefficient < −0.3 or >0.3 for both genes of each pair were considered. To analyze the functional roles of these genes, the most relevant biological processes (BP) of Gene Ontology (GO) terms were identified using the ShinyGO tool v. 0.77 (http://bioinformatics.sdstate.edu/go/) (Ge, Jung, and Yao, 2020). Pathways with a minimum of ten genes and a False Discovery Rate (FDR) < 0.05 were considered. The ten most significant pathways were selected by FDR and sorted by fold enrichment. Subsets of genes with similar BP were combined, and the DiVenn tool (https://divenn.tch.harvard.edu) (Sun et al., 2019) was used to identify subsets of upregulated and downregulated genes that are common between combinations of at least three pairs of genes. The research workflow presented in Figure 1 summarizes the methodology used.

## 3 Results and discussion

### 3.1 Identification of redox and Ca^2+^-related genes potentially relevant in BC

**FIGURE 2:**
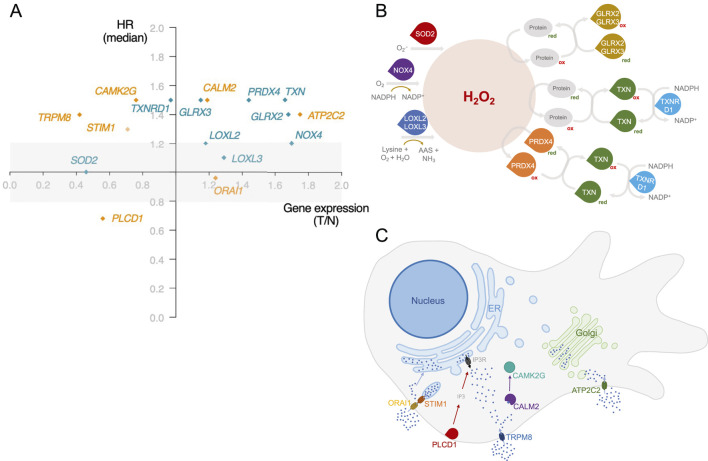
Identification of dysregulated redox and Ca^2+^-related genes associated with BC patient survival. **(A)** Gene selection was represented based on the gene expression ratio between Tumour and Normal tissues (T/N), and its impact on patient survival using a hazard ratio (HR) applying a median as a cut-off. Redox-related genes are represented in green and Ca^2+^-related genes in orange. The selection criterion of statistically significant impact on patient survival was based on median cut-off and HR < 0.8 and >1.2. Exceptionally, *LOXL3* selection was based on the quartile cut-off for HR calculation. Significance level: p < 0.05. Simplified schematic representation of the **(B)** involvement of the redox-related genes identified in this work to H_2_O_2_ metabolism and of the **(C)** function and subcellular localization of the proteins encoded by genes identified in this work related to Ca^2+^ homeostasis. Information regarding protein function was obtained from The Protein Atlas.

BC development is intricately tied to increased oxidative stress and alterations in Ca^2+^ fluxes. These alterations play a central role in cellular processes driving cancer progression (Hempel and Mohamed, 2017; Görlach et al., 2015; Reczek and Chandel, 2018). However, only a few studies explore the interplay between the simultaneous expression dysregulation of cellular redox and Ca^2+^ homeostasis genes. The gene expression analysis and the literature search identified 39 redox-signalling-related genes and 47 Ca^2+^-related genes considered relevant for BC progression and/or patient survival. Values corresponding to gene expression and HR are summarized in Supplementary Table S1. Of these, six redox-related genes - *GLRX2*, *GLRX3*, *LOXL3*, *PRDX4*, *TXN,* and *TXNRD1 -* and five Ca^2+^-related genes - *ATP2C2*, *CALM2*, *CAMK2G*, *PLCD1,* and *TRPM8* - met the following criteria: i) significant different expression between normal and BC tissues, ii) a significant impact on overall survival (median or quartile cutoff) and iii) HR lower or equal than 0.8 or greater or equal to 1.2. Despite *LOXL2*, *NOX4*, *SOD2*, *ORAI1,* and *STIM1*, only meeting one criterion, these genes were added due to their established roles in BC progression (Figure 2A; Supplementary Table S1).

The studies providing evidence supporting the influence of the selected genes on BC progression are summarized in Supplementary Table S2. By applying our selection criteria, several genes encoding thiol proteins emerged–Glrx2, Glrx3, Prdx4, Txn, TrxR1. These proteins contain a sulfur atom linked to a hydrogen atom in their active site, typically as part of a cysteine residue. This sulfur in thiol groups is highly reactive and is involved in redox reactions (Hu et al., 2019). While not all proteins involved in redox homeostasis are thiol-dependent, many utilize thiol groups to maintain the cellular redox balance (Eryilmaz et al., 2019). Notably, thiol-disulfide homeostasis can serve as a biomarker in BC patients due to its relevance in BC pathogenesis (Eryilmaz et al., 2019). Besides thiol-related genes, our selection includes *LOXL2*, *LOXL3*, *NOX4*, and *SOD2*. Loxl2 and Loxl3 play essential roles in extracellular matrix (ECM) remodelling by catalysing the cross-link between elastin and collagen. This role is associated with the invasion and migration of tumour cells (Ramos et al., 2022). Finally, like other Nox proteins, Nox4 acts as a generator of oxidants, contributing to tumorigenesis (Vermot et al., 2021). Interestingly, all these genes are directly implicated in H_2_O_2_ metabolism, as schematized in Figure 2B. Hydrogen peroxide is a key redox-signalling molecule. It mediates the reversible oxidation of redox-sensitive cysteine residues in enzymes and transcription factors regulating major features of cancer cell behaviour, such as apoptosis, cell cycle progression, proliferation, energy metabolism, and angiogenesis (Lennicke et al., 2015). While the importance of H_2_O_2_ is well recognized, significant gaps persist in our understanding of its precise mechanisms and implications in cancer cell fate. Our results also reflect this complexity. We have identified genes related to both H_2_O_2_ generation and detoxification. The overexpression of a certain gene, depending on the specific combination, may be associated with a better or worse prognosis. In addition, the redox-related genes identified in this work encode proteins with different organelle locations, highlighting the importance of the spatial H_2_O_2_ distribution. The formation of H_2_O_2_ gradients and its selective accumulation in a cellular area promote the oxidation of specific thiols within target proteins at this site, thus resulting in selective and localized H_2_O_2_ signalling events (Lennicke et al., 2015).

Regarding genes implicated in Ca^2+^ homeostasis, the selected genes have diverse molecular functions (Figure 2C). Four of the Ca^2+^-related genes identified (*ORAI1*, *STIM1*, *PLCD1,* and *ATP2C2*) code for proteins that can affect the levels of Ca^2+^ at two of the main intracellular stores: the ER and the Golgi apparatus. *ORAI1*/*STIM1* and *ATP2C2* facilitate the uptake of Ca^2+^ by the ER and the Golgi, respectively. Since *TRPM8* and *ATP2C2* allow the uptake of Ca^2+^ from the extracellular space to the cytosol and *PLCD1* controls the activation of IP3R, these three proteins impact the cytosolic Ca^2+^ levels that are sensed by a myriad of Ca^2+^ responsive proteins, including the *CALM2* and *CAMK2G* that were also identified. The dysregulated expression of some of these genes is often associated with cell survival migration and tumour growth (Zhao et al., 2023; Chodon et al., 2010; Xiang et al., 2010; Rodriguez-Mora et al., 2005; Robitaille et al., 2022). Additionally, *CALM2* is involved in BC cell cycle regulation (Rodriguez-Mora et al., 2005).

Despite the evidence of the individual impact of each of these genes in BC, a notable gap persists regarding their combined role in the mechanisms driving BC progression and, ultimately, on patient outcome.

### 3.2 Combined dysregulation of redox- and Ca^2+^-related genes and BC patient survival

**FIGURE 3:**
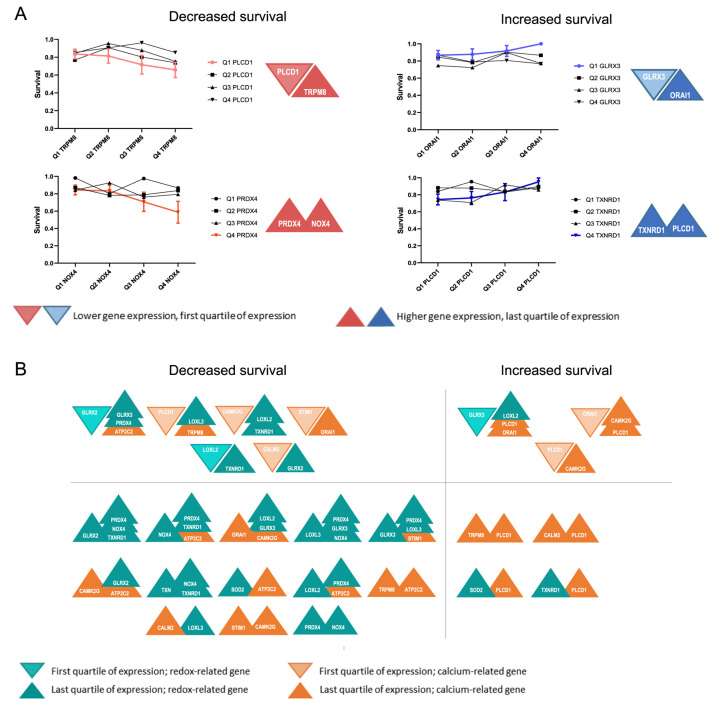
Schematic representation of the association between expression of gene pairs and BC patient survival. **(A)** Possible scenarios concerning the expression of pairs of redox and Ca^2+^ signalling genes and the survival of BC patients. **(A)** Graphs represent the cumulative overall survival proportion of patients related to the quartiles of expression of one gene associated with the quartiles of expression of the second gene. A trend was considered as significant when all four points showed an increased/decreased tendency. **(A)** Represents the four possible combinations. As an example, patients with lower expression of *PLCD1* and higher expression of *TRPM8* have a worse prognosis than patients with lower expression of both genes; Increased expression of *PRDX4* and *NOX4* is associated with decreased survival. In the opposite direction, Patients with lower expression of *GLRX3* and higher expression of *ORAI1* have a better prognosis than those with low expression of both genes, and the increased expression of *TXNRD1* and *PLCD1* is associated with increased survival. **(B)** The diagram summarizes the cases where an association was found between the combined expression dysregulation of pairs of genes involved either in Ca^2+^ (orange) or redox (green) homeostasis and the overall survival of BC patients.

To evaluate how the prognosis of BC patients correlates with the combined expression of pairs of the selected genes, the cumulative survival proportion was calculated. Considering only the first (Q1 – high expression) and last quartiles (Q4 – low expression) of gene expression, four scenarios are possible: (i) decreased patient survival is associated with lower expression of one gene and higher expression of the other (Figure 3A) or (ii) with higher expression of both genes (Figure 3A); (iii) increased patient survival is associated with lower expression of one gene and increased expression of the other (Figure 3A) or (iv) with higher expression of both (Figure 3A). Using cumulative survival proportion, the patient outcome was determined for the all combinations of genes (Figure 3B).

The gene combinations where altered expression was associated with differences in the survival of patients are depicted in Figure 3B. This approach identified 46 combinations of gene expression profiles with altered BC patient survival. Of those, 36 combinations are associated with reduced survival, while 10 are associated with increased survival. *PLCD1*, *GLRX3*, and *PRDX4* were the genes most frequently found to correlate with BC patient survival when in combination with other genes in our study, thus suggesting their importance in BC progression. The effect of most of these combinations of gene expression dysregulation in BC has not been experimentally assessed, and no mechanism is described.

Despite the complexity of redox and Ca^2+^ signalling in cancer, available mechanistic data suggest potential interactions that influence patient outcomes when both pathways are dysregulated. Recent findings indicate that the interplay between redox-regulating genes (*GLRX2*, *GLRX3*) and Ca^2+^-handling genes (*ATP2C2*, *ORAI1*, *PLCD1, TRPM8*) fosters a pro-tumorigenic environment, enhancing cancer cell survival, proliferation, and metastasis. Our findings suggest mechanistic hypotheses linking these gene pairs to BC progression, emphasizing redox-Ca^2+^ cross-talk and its downstream survival pathways.

For instance, *GLRX2* downregulation disrupts mitochondrial redox balance, reducing apoptosis (Franco and Cidlowski, 2009), while *ATP2C2* upregulation promotes Store-Independent Ca^2+^ Entry (SICE), activating the MAPK pathway and supporting tumor proliferation (Feng et al., 2010). Additionally, loss of GLRX2 weakens mitochondrial ROS detoxification, indirectly enhancing *ATP2C2*-mediated Ca^2+^ influx, which buffers oxidative stress and sustains pro-survival signaling. *GLRX3* overexpression facilitates NF-κB activation, promoting inflammatory and tumorigenic transcriptional programs (Qu et al., 2011), while *ORAI1* upregulation amplifies Ca^2+^ influx, sustaining NF-κB activity (Davis et al., 2012), reinforcing tumor cell survival and invasion. Interestingly, some gene interactions appear to have paradoxical tumor-suppressive effects. The low expression of a pro-survival gene like *GLRX3* (Qu et al., 2011) coincides with the high expression of tumor suppressors like *PLCD1* (Shao et al., 2017), and the balance may tip towards cell cycle arrest and apoptosis. Furthermore, *TRPM8* upregulation and *PLCD1* signaling alterations promote Ca^2+^ influx (Shao et al., 2017; Huang et al., 2020), which may induce ER stress-driven apoptosis.

### 3.3 Gene ontology analysis

**FIGURE 4:**
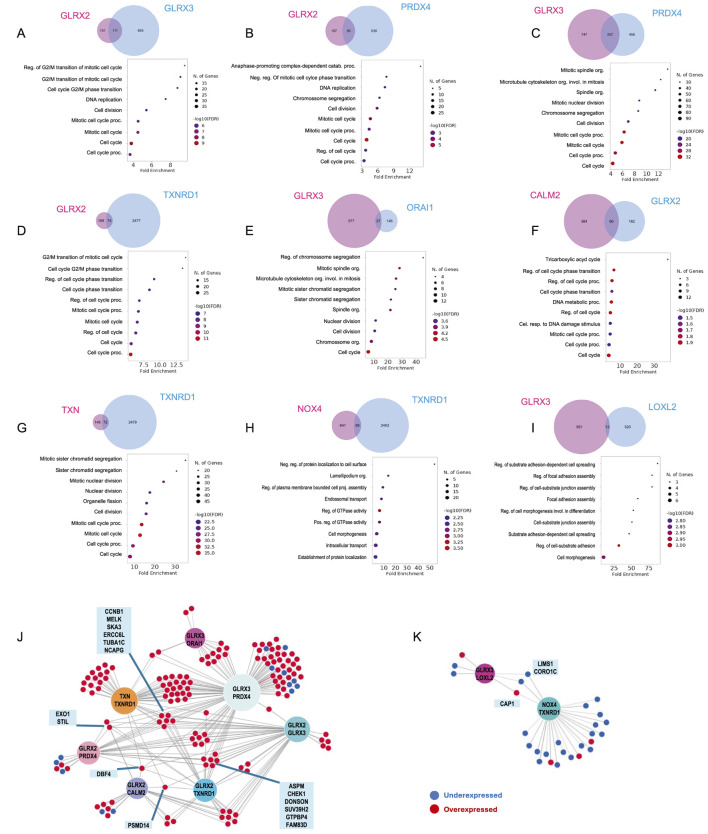
Biological process associated with co-dysregulated genes. Gene ontology enrichment analysis of genes with expression correlated to both elements of the pair identified using Venn´s diagrams. These were organized in two groups according to the biological processes identified: **(A–G)** cell cycle and **(H,I)** adhesion and cell projection related genes Associations between co-expressed genes associated with **(J)** cell cycle regulation or **(K)** adhesion and projection. Overexpressed genes are coloured red, while under-expressed genes are coloured blue. Genes overlapping between more than three pairs are highlighted.

Genes with expression positively or negatively correlated with the sets of gene combinations were identified using UALCAN. Genes correlated with both genes of each pair from Figure 3B were identified. The biological processes associated with these groups of genes were determined by enrichment analysis. Using this approach, 9 gene pairs yielded more than 10 correlated genes (Figure 4, *GLRX3. PRDX4,* n = 257; *GLRX2*. *GLRX3,* n = 111; *GLRX2*. *PRDX4,* n = 85; *CALM2*. *GLRX2,* n = 60; *GLRX2*. *TXNRD1,* n = 74; *TXN*. *TXNRD1,* n = 72; *GLRX3*. *ORAI1,* n = 27; *NOX4*. *TXNRD1,* n = 88; *GLRX3*. *LOXL2,* n = 13). Functional enrichment analysis based on Biological Processes - GO of correlated genes was performed. For several pairs, there was a significant enrichment in genes belonging to two main biological processes: i) cell cycle regulation (Figures 4A–G), and ii) adhesion and cell projection (Figures 4H–I). The GO analysis of genes co-dysregulated with *GLRX2*. *GLRX3*, *GLRX2*. *PRDX4*, *GLRX3. PRDX4, GLRX2*. *TXNRD1*, *GLRX3*. *ORAI1, CALM2*. *GLRX2*, or *TXN*. *TXNRD1* strongly suggests their association with processes related to cell cycle regulation (Figures 4A–G). The overlap of the genes presented in this enriched combination (Figure 4J) revealed that most of these genes are upregulated in BC, and some genes are common to multiple gene pairs.

The second identified cluster is related to the enrichment of *NOX4*. *TXNRD1, GLRX3*. *LOXL2* pairs. The results suggest a potential association of these genes with adhesion and cell projection mechanisms, such as regulating GTPase activity, cellular component biogenesis, and cell-substrate adhesion. The overlap of enriched genes (Figure 4K) revealed that most are downregulated in BC patients. Cell cycle (dys)regulation and adhesive properties of cancer cells directly impact cancer hallmarks and, consequently, cancer progression (Hanahan and Weinberg, 2000). The correlation found between the identified pairs of genes and these processes supports the future mechanistic exploration and validation of these combinations of genes in BC progression-related processes.

## 4 Conclusion

Several Ca^2+^ and redox-related genes were suggested as potential biomarkers or drug targets in BC. Our approach unravels potentially relevant gene pairs involved in Ca^2+^ and/or redox homeostasis and signalling, offering a more comprehensive understanding of their roles. Examining gene pairs can reveal interactions and co-regulatory mechanisms that might be missed when dysregulation of individual genes is studied. This approach may allow the identification of synergistic effects and compensatory pathways, which can lead to more precise biomarker identification and the development of combination therapies. The main mechanistic conclusions drawn from this work are based on bioinformatics analysis and pave the way for further *in vitro* and *in vivo* validation and would greatly benefit from a deeper exploration of the biological mechanisms sustaining our findings.

While our study provides significant insights into the roles of redox and Ca^2+^-related genes in BC progression, it has some limitations. First, our analysis relies on mRNA expression data from publicly available datasets, which may limit the generalizability of our findings. Given the heterogeneity of breast cancer, stratification by breast cancer subtype or tumor grade would be informative. However, the limited number of cases in certain subgroups precludes statistically robust analyses. Second, the association between protein and mRNA levels should be assessed before any identified gene is further explored as a potential therapeutical target.

By examining Ca^2+^ and redox-related gene pairs, it may be possible to uncover novel functional relationships and network dynamics that provide a deeper insight into the molecular complexity of BC, ultimately leading to more effective and personalized treatment strategies. This innovative perspective advances the field of precision oncology by contributing to highly targeted and individualized therapeutic approaches using combinations of drugs aimed at various proteins simultaneously.

## Data Availability

The datasets analysed for this study can be found at the The Cancer Genoma Atlas data portal (https://portal.gdc. cancer.gov/). The datasets generated for this study can be found at https://doi.org/10.5281/zenodo.12725287 and at https://doi.org/10.5281/zenodo.12701468.
