# Supplementary material for: Association between dysregulated expression of Ca2+ and ROS-related genes and breast cancer patient survival
Source: Front Bioinform. 2025 Sep 22;5:1633494. doi: 10.3389/fbinf.2025.1633494 (PMC12498015; doi:10.3389/fbinf.2025.1633494)
Supplement: Supplementary file 1 [file DataSheet1.pdf]

Supplementary Material

1 Supplementary Figures and Tables

Supplementary Table 1 – Tumour versus Normal tissue expression and Hazard Ratio for Ca^{2+} and Redox-related genes. Gene expression, differential gene expression (tumoral versus normal), and survival outcomes related to redox (A) and Ca^{2+}-related (B) genes. Utilizing The Cancer Genome Atlas (TCGA) data, the gene expression data was sourced from the UALCAN database (https://ualcan.path.uab.edu/). For patient survival analysis. GEPIA2 was employed.

A<br>Gene	Gene Expression (Tpm)								Overall Survival (Median)		Overall Survival (Quartil)	
	Normal			Tumoral			T/N	p(N/T)	HR(high)	p(HR)	HR(high)	p(HR)
	Q1	Med	Q3	Q1	Med	Q3	(Med)					
**LOX**	14358	25862	41660	9976	20246	35135	0.78	2.78E-01	1.20	0.200	1.30	0.230
**LOXL1**	25410	40784	58432	54412	91240	143813	2.24	1.00E-12	1.10	0.680	0.91	0.690
**LOXL2**	16021	23071	29787	16813	27233	43309	1.18	9.99E-16	1.20	0.210	1.40	0.170
**LOXL3**	**2194**	**2768**	**3906**	**2441**	**3579**	**5095**	**1.29**	**6.70E-11**	**1.10**	**0.540**	**1.80**	**0.009**
**LOLX4**	7957	10187	15207	692	1337	2404	0.13	1.62E-12	0.95	0.760	0.72	0.180
**UCP2**	83626	105074	142355	93239	145968	222239	1.39	1.62E-12	1.20	0.220	1.20	0.350
**MAP3K1**	15049	18663	24306	5462	8450	12087	0.45	1.62E-12	1.10	0.640	0.92	0.750
**PRDX1**	412 39	462701	545518	635081	820458	1116 35	1.77	1.00E-12	1.30	0.160	1.40	0.100
**PRDX2**	241258	271424	323250	319135	402806	511942	1.48	1.00E-12	1.20	0.310	1.50	0.110
**PRDX3**	179225	198523	228501	145190	196503	253349	0.99	2.51E-01	1.40	0.563	1.50	0.100
**PRDX4**	**98011**	**117045**	**134269**	**128935**	**168163**	**222397**	**1.44**	**1.62E-12**	**1.50**	**0.009**	**1.70**	**0.019**
**PRDX5**	184064	218623	245283	221585	283402	360186	1.30	1.00E-12	1.10	0.570	1.40	0.150
**PRDX6**	216753	272348	378685	190540	238652	296428	0.88	2.19E-07	1.40	0.062	1.40	0.140
**TXN**	**391749**	**475845**	**577699**	**616945**	**789196**	**1033313**	**1.66**	**1.00E-12**	**1.50**	**0.021**	**1.50**	**0.059**
**TXNRD1**	**43139**	**50475**	**58010**	**33767**	**49123**	**71812**	**0.97**	**2.27E-05**	**1.50**	**0.023**	**1.70**	**0.015**
**TXN2**	97198	105652	114878	76310	94842	114856	0.90	4.62E-07	1.30	0.170	1.10	0.600
**TXNRD2**	11246	15240	17325	11894	16453	22604	1.08	1.66E-12	1.30	0.072	1.30	0.320
**TXNIP**	939658	1243701	1642085	18241	276418	424679	0.22	1.00E-12	0.98	0.880	0.95	0.840
**GLRX**	27031	34522	46920	25661	39554	61328	1.15	1.29E-07	0.99	0.960	0.90	0.650
**GLRX2**	**12257**	**14275**	**16743**	**18440**	**23986**	**31331**	**1.68**	**1.62E-12**	**1.40**	**0.035**	**1.40**	**0.130**
**GLRX3**	**46992**	**50768**	**55222**	**48921**	**58328**	**71276**	**1.15**	**1.62E-12**	**1.50**	**0.019**	**2.60**	**0.00016**
**GLRX5**	656582	60115	65893	61043	74144	90059	1.23	1.00E-12	1.10	0.410	1.10	0.760
**GSR**	16921	21407	26494	17411	29513	47238	1.38	1.62E-12	1.20	0.270	1.40	0.140
**GPX1**	230273	315742	412515	255733	327972	418808	1.04	9.89E-01	0.96	0.780	0.75	0.230

GPX3	199548	571757	1158976	21391	37299	68213	0.07	4.77E-15	0.99	0.970	1.00	0.840
GPX4	292989	415664	659066	275789	371489	480788	0.89	1.48E-03	0.92	0.630	0.77	0.250
GPX7	13952	16648	20111	9479	16267	24303	0.98	2.47E-07	0.78	0.140	1.20	0.460
GPX8	12042	15650	20270	11603	19288	28368	1.23	1.11E-16	1.30	0.150	1.40	0.120
MPO	128	254	357	27	61	107	0.24	5.60E-15	0.98	0.900	1.20	0.520
CAT	131996	179536	281433	46266	62203	85727	0.35	1.62E-12	0.88	0.450	0.91	0.670
**SOD1**	**300608**	**336732**	**385995**	**332818**	**413668**	**512191**	**1.23**	**1.62E-12**	**1.40**	**0.025**	**1.10**	**0.680**
SOD2	370650	455919	620360	147069	210030	324541	0.46	1.62E-12	1.00	0.910	0.78	0.300
SOD3	50448	92925	147110	4868	9472	18891	0.10	1.62E-12	0.88	0.440	0.72	0.170
NOX1	0	14	31	11	3	65	0.21	1.00E-12	1.00	0.990	0.84	0.530
CYBB	13077	21907	29575	9600	17538	30197	0.80	9.23E-01	1.00	0.800	1.10	0.820
NOX4	710	1901	5094	1758	3226	5179	1.70	5.99E-02	1.20	0.300	1.30	0.220
NOX5	91	149	224	57	157	393	1.05	7.56E-11	1.20	0.240	1.20	0.350
DUOX1	1393	2865	4365	539	1192	2613	0.42	5.21E-02	1.10	0.390	1.40	0.110
DUOX2	162	302	470	3	65	138	0.22	2.47E-08	0.92	0.600	1.00	0.880

B Gene	Gene Expression (Tpm)								Overall Survival (Median)		Overall Survival (Quartil)	
	Normal			Tumoral			T/N	p(N/T)	HR(high)	p(HR)	HR(high)	p(HR)
	Q1	Med	Q3	Q1	Med	Q3	(Med)					
TRPV1	3154	4162	5433	2341	3465	5132	0.83	0.125	0.96	0.800	0.96	0.800
TRPV2	4236	6397	9588	4214	6451	9827	1.01	0.649	0.97	0.870	0.80	0.370
TRPV4	2677	4742	6809	1483	2619	3977	0.55	0.091	0.87	0.410	0.98	0.930
TRPV6	3802	7531	11352	562	2073	6153	0.28	0.930	0.98	0.920	1.20	0.460
TRPA1	12	26	41	29	73	208	2.81	0.000	0.98	0.880	0.94	0.770
TRPC1	3966	5110	6158	953	1699	2695	0.33	0.000	1.10	0.500	1.10	0.550
TRPC3	68	123	183	56	106	176	0.86	0.003	0.94	0.690	0.80	0.390
TRPC6	2550	3593	4447	621	997	1585	0.28	0.000	1.20	0.370	1.00	0.900
TRPM7	20924	25538	30410	14951	20539	26694	0.80	0.000	1.20	0.270	1.40	0.200
**TRPM8**	**23**	**67**	**123**	**9**	**28**	**62**	**0.42**	**0.000**	**1.40**	**0.038**	**1.50**	**0.065**
**ORAI1**	**16115**	**19758**	**23120**	**18887**	**24481**	**32225**	**1.24**	**0.000**	**0.96**	**0.780**	**1.30**	**0.270**
ORAI2	3202	4396	5278	5537	7516	9727	1.71	0.000	1.00	0.910	0.91	0.650
ORAI3	17727	20593	22543	16350	23637	32609	1.15	0.000	1.00	0.900	1.10	0.720
**STIM1**	**41579**	**47098**	**52490**	**25102**	**33289**	**43191**	**0.71**	**0.000**	**1.30**	**0.130**	**1.20**	**0.380**
STIM2	13799	17532	20916	8771	11623	14592	0.66	0.000	1.00	0.990	0.97	0.900
SARAF (SOCE)	271149	319484	363630	178182	246665	348529	0.77	0.001	1.20	0.380	0.96	0.840
ATP2B1 (PMCA1)	16327	22225	27611	10017	14389	19053	0.65	0.000	0.93	0.640	1.40	0.140
ATP2B2 (PMCA2)	119	173	232	25	52	103	0.30	0.727	0.92	0.620	1.00	0.880

**PMCA4**	54077	75796	109030	26753	40121	56093	0.53	0.000	0.83	0.250	1.10	0.820
**ATP2A3 (SERCA3)**	12687	16250	21303	17979	32532	58117	2.00	0.000	1.30	0.110	1.10	0.750
**CALM1 (CAM)**	118722	158565	183047	99194	124711	154815	0.79	0.000	1.30	0.076	1.40	0.170
**CALM2 (CAM)**	**616842**	**707061**	**806536**	**685901**	**840372**	**1017677**	**1.19**	**0.000**	**1.50**	**0.023**	**1.60**	**0.031**
**CALM3 (CAM)**	138388	154589	171119	151077	185510	227464	1.20	0.000	1.10	0.490	1.30	0.240
**CAMKIA**	16298	26839	41388	9134	12539	16533	0.47	0.000	1.30	0.100	0.92	0.730
**CAMKID**	2541	3273	4325	2179	3632	5906	1.11	0.000	1.20	0.290	1.50	0.120
**CAMK2B**	509	1127	2407	255	765	1971	0.68	0.539	0.78	0.140	0.67	0.098
**CAMK2D**	16424	18360	20631	5801	9864	14530	0.54	0.000	1.20	0.220	1.40	0.140
**CAMK2G**	**18598**	**21459**	**23774**	**12826**	**16391**	**20401**	**0.76**	**0.000**	**1.50**	**0.024**	**1.80**	**0.011**
**CAMKK1**	4462	6175	7355	3467	4927	7131	0.80	0.856	1.40	0.064	1.60	0.051
**CAMKK2**	33277	36973	40805	23958	30963	37575	0.84	0.000	0.99	0.960	0.94	0.780
**ATP2C2 (SPCA2)**	**2170**	**4422**	**7401**	**4267**	**7752**	**13289**	**1.75**	**0.000**	**1.40**	**0.038**	**1.40**	**0.130**
**ITPR1 (IP3R1)**	33242	51434	71372	10247	19081	33096	0.37	0.000	1.30	0.150	1.30	0.260
**ITPR2 (IP3R2)**	16249	23623	32550	6023	10797	18229	0.46	0.000	0.88	0.440	0.86	0.520
**ITPR3 (IP3R3)**	9923	14708	19137	14897	21973	29633	1.49	0.000	1.10	0.630	1.10	0.680
**FAM38A (PIEZO1)**	39669	48170	60729	34522	48039	67265	1.00	0.002	1.00	0.960	0.96	0.880
**FAM38B (PIEZO2)**	2938	5737	8841	1781	4268	8798	0.74	0.000	1.10	0.600	1.00	0.850
**TPCN1 (TPC1)**	31915	35567	40969	17376	24741	33427	0.70	0.000	1.20	0.180	1.50	0.081
**TPCN2 (TPC2)**	5577	6553	7563	3848	5115	6759	0.78	0.474	1.00	0.940	1.00	0.840
**CAPN1**	73037	101480	116306	96634	121296	154653	1.20	0.000	1.20	0.230	1.50	0.083
**CAPN2**	116136	140962	160238	84923	124747	181214	0.88	0.454	1.20	0.230	1.40	0.130
**CAST**	136079	165625	201340	82317	111466	144311	0.67	0.000	1.10	0.440	1.00	0.920
**CCDC109A (MCU)**	12035	13856	15405	11524	14814	18916	1.07	0.001	1.30	0.140	1.10	0.640
**UCP2**	83626	105074	142355	93239	145968	222239	1.39	0.000	1.20	0.220	1.20	0.350
**UCP3**	625	872	1142	471	737	1045	0.85	0.330	0.78	0.130	0.78	0.310
**PLCD1**	**14102**	**16795**	**18813**	**6473**	**9387**	**13175**	**0.56**	**0.000**	**0.68**	**0.019**	**0.63**	**0.053**
**NTSR1**	9	19	57	7	23	56	1.21	0.000	1.20	0.360	1.20	0.470
**TMBIM4**	6442	76318	84314	59224	82102	110009	1.08	0.004	1.00	0.820	1.10	0.750

Supplementary Table 2 – Summary of studies related to the impact of the selected redox- and Ca2+-related genes in BC outcome. This table summarizes the primary functions of selected redox- and Ca^{2+}-related genes and various studies reporting their impact on breast cancer.

Gene	Main gene function	Effect in BC *
Glutaredoxin-2 (***GLRX2***)	Glutathione-dependent oxidoreductase enzymes	10.1096/fasebj.23.1_supplement.LB253<br>10.1074/jbc.M408011200
Glutaredoxin-3 (***GLRX3***)		10.1172/JCI43144
Lysyl oxidase like 2 (***LOXL2***)	Copper-dependent amine oxidases that catalyse the formation of crosslinks in collagens and elastin in the extracellular matrix	PMID: 12154058<br>10.1007/s10549-013-2662-3<br>10.1002/emmm.201100156
Lysyl oxidase like 3 (***LOXL3***)		10.1038/s41388-022-02258-1
NADPH Oxidase 4 (***NOX4***)	Catalytic subunit the NADPH oxidase complex. Acts as an oxygen sensor and catalyzes the reduction of molecular oxygen to various reactive oxygen species	10.4161/cbt.10.3.12207<br>10.1016/j.bcp.2013.05.011
Peroxiredoxin-4 (***PRDX4***)	Thiol-specific peroxidase that catalyzes the reduction of hydrogen peroxide and organic hydroperoxides to water and alcohols, respectively	10.1016/j.canlet.2015.03.012<br>10.23937/2378-3419/3/2/1053
Superoxide dismutase 2 (***SOD2***)	Oxidoreductase that converts superoxide anion to hydrogen peroxide and diatomic oxygen	10.1016/j.freeradbiomed.2014.08.026<br>10.1016/j.freeradbiomed.2008.09.005
Thioredoxin (***TXN***)	Catalyzes dithiol-disulfide exchange reactions. Plays a role in the reversible S-nitrosylation of cysteine residues in target proteins	10.1016/j.redox.2015.12.004<br>10.1186/bcr2599
Thioredoxin reductase 1 (***TXNRD1***)	Reduces disulfide protein thioredoxin to its dithiol-containing form	10.1186/bcr2599<br>10.1038/srep36860
ATPase Secretory Pathway Ca^{2+} Transporting 2 (***ATP2C2***)	ATP-driven pump that supplies the Golgi apparatus with Ca^{2+} and Mn^{2+} ions. Interacts with *ORAI1*	10.1016/j.cell.2010.08.040<br>10.1016/j.redox.2022.102240
Calmodulin 2 (**CALM2**)	Mediates the control of enzymes, ion channels, aquaporins and other proteins through calcium - binding	10.1007/s10549-008-0097-z<br>10.3390/proceedings2251550
Ca^{2+}/Calmodulin-stimulated protein kinase II gama (***CAMK2G***)	Ca^{2+}/calmodulin-dependent protein kinase	10.1038/srep33132
Ca^{2+} release-activated Ca^{2+} modulator 1 (***ORAI1***)	Ca^{2+} release-activated Ca^{2+} channel which mediates SOCE activation by the Ca^{2+} sensor STIM1	10.1016/j.ccr.2008.12.019<br>10.1016/j.bbrc.2011.07.025
Stromal interaction molecule 1 (***STIM1***)	Ca^{2+} sensor in the ER and upon Ca^{2+} depletion, translocate from the ER to the PM where it activates ORAI1	
1-Phosphatidylinositol-4.5-bisphosphate phosphodiesterase delta-1 (***PLCD1***)	Catalyzes the hydrolysis of PIP2 into the second messengers DAG and IP3	10.18632/oncotarget.16072<br>10.4161/cbt.10.5.12726
Transient receptor potential melastatin 8 (***TRPM8***)	Receptor-activated non-selective cation channel	10.1007/s13277-014-2077-8<br>10.1186/1471-2407-10-212
